# Anamnesis of Sycosis*Translated from *Allg. Hom. Zeitung*, 65, 100, by Carroll Dunham, M. D. Republished now by special request.

**Published:** 1883-11

**Authors:** C. Von Bœnninghausen

**Affiliations:** Munster


### ANAMNESIS OF SYCOSIS.*

Dr. C. Von Bœnninghausen, Munster.

Every homœopathic physician, the new as well as the old homœopath, understands and sets a due value upon that which we understand by the word *Anamnesis.*

The scope of this Anamnesis, it is well known, is not limited to mere external sources of injury, such as a fall, a blow, a contusion, a luxation, burns, getting wet and the like; nor to previous illness, like measles, scarlatina, etc.; nor, finally, to various emotions; nor to all the other manifold inducing causes which are often followed by serious maladies;—we avail ourselves of it in like manner, and with just as decided results, in the prophylactic treatment of contagious pestilences, without needing to await the outbreak, or, at all events, the widespread prevalence of the disease, in so far as this—that a fully developed case of the disease occurring in our vicinity enables us to select with certainty the appropriate remedy, and this remedy serves as the surest prophylactic against the contagion of the same malady.

If we admit the justice of what has been said, and uniform experience compels us to admit it, no person of sound reasoning powers can fail to see the great inconsistency of denying in chronic diseases what we admit and have proved to be true in acute diseases. And yet the much condemned and derided theory (as it is called) of the founder of Homœopathy concerning the three miasms (Psora, Syphilis, and Sycosis) is nothing else but a logical application of the *Doctrine of the Anamnesis* to chronic diseases, as is expressed in the most explicit words in the *Organon*, § 5 and § 206, 5th edition.

---

* Translated from *Allg. Hom. Zeitung*, 65, 100, by Carroll Dunham, M. D. Republished now by special request.

It is incomprehensible, then, how it has happened that this fact has been so completely overlooked, if, indeed, other and not very praiseworthy motives have not come into play. For all the fine phrases about exclusively following the fundamental principle of the homœopathic mode of cure are of no avail to deceive the experienced practitioner, and to persuade him that he will in all cases be able to make the most exactly fitting choice of a remedy, by the aid of even page upon page of symptons, in which, after all, nothing of a therapeutic-characteristic nature is to be found!

I will not deny the possibility that, in addition to the three anamnestic indications already named and in addition to the true drug cachexies, there may be one or several other miasms to which a similar chronic influence upon health may be ascribed. But the existence of such a miasm has not hitherto been satisfactorily demonstrated, and it must be left for future investigations to determine its existence.

If, however, we adhere to that which we have already before our eyes, and make an unprejudiced estimate of the results of the homœopathic practice in the treatment of chronic diseases in accordance with the Hahnemannian doctrine, it cannot be denied that the achievements of our younger science surpass those of her older sister.

The acute investigations and their results, which our colleague Wolf has laid before us in his *Homœopathische Erfahrungen* (part 2–5), and in which the domain of Sycosis receives so general an expansion, explain to our complete satisfaction the failure to cure many cases that belong to this domain, because the true Anamnesis of those cases was not known and could not be taken advantage of. But since now the identity of Variola with Sycosis seems to be sufficiently demonstrated and the great extension of this miasm through the process of inoculation is placed beyond a doubt, the treatment of numerous chronic diseases, of which Psora had been hitherto erroneously regarded as the Anamnesis, has taken quite another aspect and has become much more certain.

On the other hand, we must not disguise the truth that out of the facts mentioned a new practical difficulty arises, viz.: that we have as yet no certain means of knowing accurately whether the one or the other miasm dominates. For a large majority of the symptoms in chronic diseases are such as are found among the symptoms of all the three miasms, and we lack, up to the present time, that sifting and separation of them which are indispensable, inasmuch as many of them belong exclusively to the one or the other of the miasms, and for that reason would serve for the establishment of the Anamnesis which is of so very great importance.

Inasmuch as I have probably used Thuja longer and more extensively than any living homœopathist, and was the first to discover its almost specific curative virtues in small-pox, in diabetes mellitus, in certain malignant aphthæ in children, in volvulus, etc., I shall not be considered presumptuous if I venture to offer in the following pages a contribution toward a list of those symptoms which are common to Thuja and the widespread Sycosis. They may enable us in many cases to recognize the anamnestic miasm in question, and thus enable us from the beginning to give a corresponding direction to the treatment.

In doing this, however, less attention is to be paid to that which, even though it be more prominent, is yet common to two or three of the miasms, than to that which is exclusively peculiar to one of them, and is found in connection with it alone.

Such a separation and isolation of the special phenomena makes it apparent that the entire picture of the disease must be in and of itself a very incomplete and defective one, but it will so much the more distinctly bring into relief before the eye those symptoms which enable us to recognize the anamnestic miasm.

In this work I have naturally, first of all, compared with one another the chief remedies for the simple forms of the three miasms above named (Sulphur, Mercurius, and Thuja), and have rejected all which the first two remedies presented in common with the last.

But in the course of this comparison, it appears at the same time that several of those remedies which Hahnemann enumerated among the Antipsorics may with equal propriety be included also among the Antisycotic remedies. The names of these remedies are attached, in parenthesis, to the symptoms under which they belong, and in this way the array of antisycotic remedies is considerably enlarged, a matter of especial importance when we have to deal with complications of the fundamental miasms—in which case we are enabled to attain our end with one chief remedy alone.

When a similar comparison shall have been made of the chief antisyphilitic remedy (Mercurius), and when the picture of Psora, as Hahnemann has given it in the *Chronic Diseases* (1, 58, 67), shall have been reduced to its own peculiar characteristics by the exclusion of the symptoms of Mercury and of Thuja, the treatment of chronic diseases will, I believe, have been rendered thereby much easier and more certain. I venture to hope, therefore, that the foregoing observations, as well as the following modest experiment, may not entirely fail of a favorable reception, but that rather the whole may be elucidated and expanded on the basis of more extended experiences.

### SPECIAL SYMPTOMS OF THUJA.

MIND:

*Fixed Ideas*, that a stranger is always by his side. (Anac.)

—that mind and body are separated. (Anac.)

—*that the body, and especially the limbs, are made of glass and are very fragile* (?).

VERTIGO:

*Vertigo on closing the eyes*, relieved immediately on opening them again. (Apis, Lach.)

INTERNAL HEADACHE:

5. Feeling of numbness and emptiness, only in the vertex (?).

Pain in the vertex as if a nail were driven in. (Hell., Staph.)

The headache is *ameliorated* generally by *motion in the open air*, by *looking upward*, and by *bending the head backward*. (Apis, Bell., Rhus.)

EXTERNAL HEAD:

Painfulness of the scalp *when touched, and of the parts on which one lies*. (Nitr. acid, Rhus.)

He desires always to have the head closely wrapped up. (Lach., Rhus.)

EYES:

10. Suffusion of the eyes, especially in the open air; the tears do not flow down, but remain in the eye. (Caust., Nit. acid, Sep.)

Inflammatory relaxation of the inner surface of the lid. (Rhus.)

VISION:

On one side, near the eye, an appearance in darkness as of flashes of lightning or of sparks, in daylight as of dark drops (?).

Objects always appear smaller. (Plat., Stram.)

HEARING:

Noise in the ear, as of boiling water. (Dig.)

NOSE:

15. *Warts on the nose.* (Caust.)
*Eruptions in the angles of the nose.* (Euphr., Rhus.)
Swelling and hardness of the alae nasi (?).

SMELL:

Odor in the nose as of herring brine or of fermenting beer. (Bell., Vit.)

FACE:

*Glowing redness of the whole face, with a fine network of blood-vessels, as if it were marbled.* (Calc. c., Carb. veg., Lyc.)
20. Eruption in the face which leaves bluish spots. (Ferr., Lachesis.)
Light brown spots (freckles) in the face. (Ant. cr., Calc., Graph., Kali, Natr., Nit. ac., Phos.)
*The skin of the face is oily.* (Natr. mur., Selen.)
*Scaling off of the skin of the face.* (Apis.)
*Distention of the veins of the temples.* (Chin., Ferr.)

LIPS AND CHIN:

25. *Flat, whitish ulcers on the inner side of the lips and in the corners of the mouth.* (Graph., Mez.)
Cracking in the maxillary articulation. (Nitric. acid, Rhus.)

TEETH:

*Crumbling of the teeth.* (Bor., Lach., Staph.)
*The roots of the teeth decay.* (Mezer.)
*The teeth become hollow upon the side, while the crown is not affected.* (Mezer., Staph.)
30. Eating, gnawing pain in the hollow teeth, aggravated by cold. (Rhus, Staph.)
Toothache from drinking tea. (Ferr., Selen.)

MOUTH:

Painful deglutition, worse on empty swallowing or when only saliva is swallowed. (Lach., Rhus.)
Gelatinous ranula. (Mezer., Nit. acid, Staph.)
Aversion to potatoes. (Alum. ?)

35. *Bad effects from tea.* (Chin., Ferr., Selen.)
—*from sugar.* (Merc., Selen.)
—*from onions.* (Lyc., Puls.)

TASTE:

Food tastes as if it were *not salt enough.* (Ars., Calc., Cocc.)

*Bread tastes dry and bitter.* (Ferr., Rhus.)

In the morning, a taste as from rotten eggs in the mouth. (Arn., Hep., Phos., Phos. acid.)

ERUCTATIONS:

Constant eructations when eating. (Nit. acid.)

NAUSEA:

40. *Fatty vomiting.* (Ars., Mezer., Iod., Nux vom.)

STOMACH;

Induration of the stomach. (Mezer.)

*Drink passes into the stomach with a noise (?).*

ABDOMEN:

A drawing inward of the epigastrium. (Apis, Staph.)

ABDOMINAL WALLS:

Soreness of the umbilicus. (Rhus.)

45. Herpes zoster. (Graph., Rhus.)

*Yellow or brownish spots upon the abdomen.* (Sepia.)

GROINS:

*Swelling of the inguinal glands.* (Calc., Nit. acid, Rhus, Staph.)

FLATULENCE:

Like the crying of an animal in the abdomen. (Arg. ?)

STOOL:

Ineffectual desire for stool, *accompanied by erections.* (Ignat. ?)

50. *In the morning or forenoon, diarrhœa recurring at the same hour.* (Ap., Sabad.)

Fat, oily stools. (Caust.)

ANUS:

*Offensive sweat at the anus and in the sulcus between the nates (?).*

Painful contraction of the anus at stool. (Staph.)

*Condylomata at the anus.* (Nit. acid, Sabina, Staph.)

PERINEUM:

55. *Sweat of the perineum.* (Alum., Carbo an.)

Knotty puffiness and excoriations on the perineum (?).

URINE:

*Frothy urine.* (Kali, Lach., Lyc.)

*Saccharine urine.* (Chin., Phos.)

Continued dropping of the urine, after urinating. (Lach., Selen.)

GENITALS:

60. *Copious sweat upon the genitals, of a sweet, honey-like odor, and imparting a yellow stain (?).*

MENSTRUATION:

*Copious sweat before the menses.* (Verat?)

Abortion in the third month. (Apis, Sabin., Sec. corn.)

CORYZA:

*Coryza, fluent when in the open air, and dry in the chamber.* (Iod., Platina, Puls.)

Much mucus in the choanæ. (Euphras., Nit. acid, Zinc.)

Fluent coryza and sneezing give immediate relief. (Lach.)

RESPIRATION:

*Dyspnœa, as if the lungs had become adherent to the thorax.* (Mezer.)

*Shortness of breath from fullness and constriction in the hypochondria and epigastrium.* (Staph.)

*Dyspnœa from accumulation of mucus in the trachea.* (Selen.)

COUGH:

*He coughs only in the daytime;* also in the morning after rising and in the evening after lying down, but seldom at night. (Euphr., Lach., Nit. acid, Staph.)

70. *During the evening cough, after lying down, the sputa are dislodged more easily when he turns from the left side to the right.* (Kali, Lyc., Phos., Sepia.)

The sputa taste like old cheese. (Chin., Kali, Lyc.)

INTERNAL THROAT:

Swelling and feeling of obstruction in the throat. (Apis, Mezer.)

*Sensation as if there were a skin in the larynx.* (Lach., Phos.)

EXTERNAL THROAT:

Blue distended veins of the neck. (Ars, Lach.)

75. *Oily, brown skin in the nape of the neck.* (Apis, Lyc.)

INTERNAL THORAX:

Sensation of a hot rising in the thorax. (Phos.)

*Sensation as of drops falling in the chest (?).*

*Sticking in the chest, after a cold drink.* (Staph.)

Orgasm of the blood and audible palpitation of the heart. (Dig., Iod., Spig.)

80. *Anxious palpitation in the morning on waking.* (Rhus, Spig.)

External Thorax:

Blueness upon the clavicles. (Lach. ?)

*Brownish spots upon the chest.* (Lyc., Phos., Sepia.)

Back:

*Burning from the sacrum upward to the scapulæ.* (Phos., Sepia.)

Throbbing and pulsation in the back. (Bar., Lyc., Phos.)

85. Boils upon the back. (Caust., Graph., Hepar.)

Upper Extremities:

Herpes upon the elbow. (Phos., Sep., Staph.)

*Brown color of the dorsum of the hand.* (Iod. ?)

White scabby Herpes on the dorsum of the hand and on the fingers. (Lyc., Sepia.)

*Hands covered with cold sweat.* (Sepia.)

90. *Warts upon the hands.* (Lach., Nit. acid, Rhus.)

*Erysipelatous swelling of the tips of the fingers with formication in them.* (Rhus.)

*The finger-nails are distorted, crumbling, and discolored.* (Graph., Nit. acid, Silic.)

Lower Extremities:

Laxness in the hip-joints. (Apis, Calc., Staph.)

When walking, the legs feel as if they were made of wood. (Plumb., Rhus.)

95. Pain in the hip, the limb becoming longer than before. (Coloc., Rhus.)

*The skin of the extremities assumes a brown color, especially on the inner side of the thigh (?).*

*The dorsum of the foot is as it were marbled, with a network of blood-vessels.* (Caust., Lyc.)

Burning corns. (Ammon., Bar., Phos. acid, Rhus.)

*Red swelling of the ends of the toes.* (Chin., Mur. acid.)

100. *The toe-nails are brittle and distorted.* (Ars., Graph., Sabad., Sepia.)

*Offensive sweat of the toes.* (Bar., Graph., Kali., Nit. acid, Puls., Sil.)

Suppressed sweating of the feet. (Apis, Kali, Rhus, Sepia, Sil.)

General Symptoms:

Emaciation and appearance of the parts affected as if they were dead. (Ars., Carb. veg., Graph., Mezer., Plumb., Selen.)

*Frequent starting up of the trunk.* (Natr. mur., Nit. acid, Sepia.)

105. *The flesh feels as if it were beaten loose from the bones.* (Apis, Lach., Nitr. acid, Rhus.)

*Feeling of lightness in the body when walking.* (Chin., Rhus, Spig.)

*Sensation of tenderness and fragility in the body (?).*

*Cracking of the joints when they are extended.* (Lyc., Rhus.)

Abuse of Sulphur and Mercury. (Caust., Puls., Sep.)

110. Recurrence of the symptoms after the lapse of a year. (Ars.)

Aggravation in the evening and night.

Aggravation of certain symptoms about three o'clock morning and afternoon.

*Cold moisture aggravates, warm moisture relieves.*

*Eructation as well as fluent coryza with sneezing afford instant relief.*

115. *Many (internal and external) symptoms are relieved by turning from the left to the right side while lying down.*

Bad effects from the use of beer, fat, acids, sweets, tobacco, *tea*, wine, and *onions*. (Ars., Chin., Ferr., Lach., Sepia.)

BONES:

Rachitic affections of the bones.

SKIN:

Dirty-brownish color of the skin (Ferr., Iod.)

Brownish spots upon the skin. (Ant. cr., Carb. veg., Lyc., Mezer., Nit. acid, Phos., Sep.)

120. *Brown-reddish* (Nit. acid, Phos.) *or brownish-white spots.* (Ars., Phos., Sep., Sil.)

*Fine network of blood-vessels, like a marbling.* (Carb. veg., Caust., Lyc., Plat.)

*Eruption only upon the parts that are covered.* (Led.)

*Pox (vesicæ).* (Ant. cr., Ant. tart., Ars., Bell., Nit. acid, Merc., Rhus.)

Varicellæ. (Ant. cr., Ant. tart., Carb. veg., Puls., Sep.)

125. *All eruptions burn violently after cold-washing (?).*

*Condylomata, which often smell like old cheese or like herring brine.* (Calc., Graph., Hep.)

*Large, jagged, often pediculated warts, which become moist and easily bleed.* (Caust., Lyc., Nit. acid, Phos. acid, Rhus, Staph.)

*Itching, scabby herpes.* (Graph., Rhus, Sep.)

*Dry Pityriasis, throwing off whitish scales.* (Ars., Calc., Dulc., Lyc., Sep., Sil.)

130. Herpes cincinnatus. (Graph., Iod., Natr., Sep.)

*Flat ulcers with a bluish white bottom.* (Ars., Lach., Lyc., Sep., Sil.)

*The nails of the fingers and toes are distorted.* (Caust., Graph., Nit. acid, Sabad., Sil.)

Corroding itching in the skin relieved by scratching, but then succeeded by burning. (Caust., Lach., Mezer., Rhus, Sulph.)

Luxuriant growth of hair on parts not usually covered with hair (?).

SLEEP:

135. *Continued sleeplessness, with painfulness of the parts on which he lies.* (Hep.)

*Sleeplessness; visions appear as soon as he closes his eyes, but disappear when he opens them again.* (Apis? Lach.?)

Sleep comes late by reason of restlessness and heat. (Bry., Phos., Rhus.)

Anxious dreams when lying on the left side. (Lyc., Phos., Puls., Sep.)

FEVER; CIRCULATION:

Evening, orgasm of the blood.

—CHILL:

140. *Evening and night, cold creepings run often through the back.* (Ars., Puls., Rhus.)

Chill, as if dashed with cold water. (Merc., Mezer., Puls., Rhus.)

—HEAT:

*Dry heat during sleep.* (Samb.)

—SWEAT:

*Sweet sweat, smelling like honey.* (Bry.? Puls.? Selen.?)

*Sweat, imparting a brownish-yellow stain.* (Ars., Bell., Carb. an., Graph., Lach., Magn., Selen.)

145. Cadaverous exhalations from the skin (?).

General sweat, except upon the head. (Bell., Rhus, Samb.)

In the morning when walking in the open air, copious sweat chiefly on the head. (Calc.)

*Sweat most copious upon the upper part of the body.* (Carb. veg., Nit. ac., Nux v., Sec. corn., Sep., Sulph. ac.)

*Sweat, either of those parts alone which are covered* (Bell., Chin., Spig.) *or of those alone which are uncovered (?).*

150. *Sweat during sleep, ceasing at once on waking.* (Euphr., Nux v., Phos.)

From the foregoing series of symptoms, which may be regarded as comprehending perhaps the most important of the symptoms peculiar to Thuja (and to pure sycosis?) as yet known to us, it will be perceived that a more or less close relationship exists between Thuja and the following remedies:

Anac., Ant. crud., APIS, ARS., Bar., Bell., CALC., Carb. an., Carb. v., CAUST., CHIN., Euphr., FERR., GRAPH., Hep., IOD., Kali., *Lach.*, *Lyc.*, MEZER., *Nit. acid*, PHOS., Phos acid, Plat., Plumb., PULS., *Rhus*, Sabad., SELEN., *Sep.*, SIL., Spig., *Staph.*

As these remedies by this very concurrence point to a Sycotic Anamnesis, so experience has already established by numerous cases that their administration is particularly efficacious against symptoms which unquestionably originate in this source, provided always that in other respects they are selected in accordance with the fundamental principle of Homœopathy. For we very seldom succeed, in practice, in destroying by means of Thuja alone the entire sycotic miasm in all its manifold protean forms, just as rarely as we succeed with Sulphur alone in the case of psora or with Mercury alone in syphilis and its numerous sequelæ.

Still less is this to be expected when, as so often happens, complications of two or of the three miasms present themselves, a circumstance of which Hahnemann speaks in his *Chronic Diseases* (1, 115, 2d Ed.), and which, in truth, is not so rare as one might suppose.

Least of all, however, may we expect to accomplish the cure with but few remedies in cases in which many other remedies have already been used, and where, thereby, as we read in the *Organon*, § 75, "a perversion of the normal condition" has been induced such as, "when it has attained a considerable height," might, it would seem, be regarded as incurable by drugs alone and which one must therefore be prepared to subject to a very long course of treatment.

It is worthy of remark, in conclusion, that in such cases the above-named remedies, as a general thing, deserve the preference even over other remedies which in like manner produce among their effects sycotic symptoms and even condylomata (although these are mostly of a different kind).

Among the latter are: Ant. tart., Apis, BAR., Bell., Bry., CALC.,

CAUST., CHAM., DULC., EUPHR., Hep., IOD., LACH., *Lyc.*, Mezer., *Nit. acid*, NUX VOM., Petr., PHOS. ACID, RHUS, SABIN., Sec. corn., Selen., SILIC., *Staph.*, SULPHUR.

May there not perhaps be condylomata, just as there are many forms of gonorrhœa which are not strictly sycotic in their nature, and have *really* nothing in common with it? Great and apparent as is the general resemblance between these two series of remedies, there are nevertheless important differences, as well in respect of the remedies themselves as of their relative value. Meanwhile it must not be forgotten that the sycotic anamnesis in its present extension is a new product of the ever-blooming evolution of Homœopathy, and that, after the lapse of a few years, when further experiences shall have been accumulated on the subject, many changes and additions in this department may be expected.

Moreover, it must be obvious to every one that this subject is one of the greatest importance, and that we have reason to give it a careful consideration, since it is more than probable that the *pure homœopathic anamnesis* will place us on the path of success in the treatment of several chronic diseases which have hitherto proved incurable even by us. Whoever, therefore, has at heart the farther expansion of our blessed science and the alleviation of many desperate maladies under which his fellow-men are suffering, will hardly lay this communication aside condemned in advance, unheeded, or unproved.

